# Effects of black pepper and turmeric powder on growth performance, gut health, meat quality, and fatty acid profile of Japanese quail

**DOI:** 10.3389/fphys.2023.1218850

**Published:** 2023-07-11

**Authors:** O. Ashayerizadeh, B. Dastar, M. Shams Shargh, E. A. Soumeh, V. Jazi

**Affiliations:** ^1^ Department of Animal and Poultry Nutrition, Faculty of Animal Science, Gorgan University of Agricultural Sciences and Natural Resources, Gorgan, Iran; ^2^ School of Agriculture and Food Sustainability, University of Queensland, Brisbane, QLD, Australia

**Keywords:** antibiotics as growth promoters, black pepper, gut health, phytogenic, turmeric

## Abstract

In poultry production, the search for alternatives to in-feed antibiotics continues unabated. This study investigated the effects of dietary supplementation of black pepper and turmeric powder, separately or in combination, on the growth performance, gastrointestinal microbiota population, intestinal morphology, serum biochemical parameters, meat quality, and meat fatty acid profile in Japanese quails. Five hundred-day-old mixed-sex Japanese quail chicks were randomly assigned to one of five treatments: a control diet (CON); CON +0.2% antibiotic flavomycin as an antibiotic growth promoter (AGP); CON +0.5% turmeric powder (TUP); CON +0.5% black pepper powder (BPP); and CON +0.5% TUP, and 0.5% BPP (MIX). The findings showed that quail chicks fed AGP and TUP throughout the rearing period had better body weight gain (*p* = 0.007) and feed conversion ratio (*p* = 0.02) than the other treatments. The TUP, BPP, and MIX feeds reduced (*p* = 0.005) abdominal fat percentage. The MIX group had a better breast muscle water-holding capacity (*p* = 0.04) and lightness index (*p* = 0.02) and lower (*p* = 0.02) malondialdehyde concentration after 7 days of refrigerated storage. Feeding BPP, TUP, and MIX diets decreased (*p* = 0.001) serum cholesterol concentration. Quail chicks fed the CON diet showed significantly higher coliform counts in the crop and ileum (*p* < 0.001), whereas the lactic acid bacterial population was lower (*p* = 0.008) in the ileum. Birds that received the MIX diet exhibited a higher (*p* = 0.02) villus height to crypt depth ratio in the duodenum compared to the other groups. The tested feed additives increased (*p* < 0.001) villus height in the jejunum and ileum compared to other groups. Feeding the TUP, BPP, and MIX diets reduced (*p* < 0.001) total saturated fatty acid content and increased (*p* = 0.004) total polyunsaturated fatty acid concentration, where the MIX diet had the best results. Overall, the present data indicate that supplementing the basal diet with turmeric powder enhances the growth performance of Japanese quails. In some respects, such as gut health and meat quality, combining turmeric powder and black pepper powder was more effective than using them independently.

## Introduction

The use of phytogenic feed additives (PFAs) in animal feeding has gained significant attention in recent years, particularly after the ban on antibiotics as growth promoters. PFAs are plant-derived substances and are utilized to improve livestock and poultry productivity. These additives encompass a wide range of herbs, spices, and their derivatives (Windisch et al., 2008). The bioactive compounds found in PFAs, such as polyphenols, organic acids, essential oils, terpenoids, and aldehydes, are known to possess antioxidant and antibacterial properties that can have a positive impact on animal productivity (Shirani et al., 2019). In addition, PFAs can stimulate blood circulation and promote the production of digestive secretions, including mucin, amylase, lipase, trypsin protease, and bile acid (Mohebodini et al., 2021). Previous studies have shown that PFAs can impact the gut microbiome (Barbarestani et al., 2020), improve lipid metabolism (Mohebodini et al., 2019), enhance intestinal mucosal morphology, strengthen the intestinal barrier function (Oso et al., 2019), and boost the immune system of broiler chickens (Pirgozliev et al., 2019). However, our knowledge about their applications in poultry nutrition is still somewhat limited.

Turmeric (*Curcuma longa*) is a spice that belongs to the Zingiberaceae family and is widely distributed in tropical and subtropical regions of the world, particularly in Asian countries (Amalraj et al., 2017). As a traditional herbal medicine, turmeric has been used for centuries to treat various ailments, including urinary tract infections, liver disorders, inflammation, menstrual disorders, and digestive issues (Salehi et al., 2019). Both *in vitro* and *in vivo* studies have demonstrated that turmeric possesses numerous biological and pharmacological properties, such as antioxidant, antibacterial, antifungal, anti-inflammatory, and immunomodulatory effects (Strimpakos and Sharma, 2008; Mirzaei et al., 2017). The pharmacological activity of turmeric is primarily attributed to the presence of various curcuminoid compounds, including curcumin, demethoxycurcumin, and bisdemethoxycurcumin (Salehi et al., 2019).

Black pepper (*Piper Nigrum*) is another significant, widely-used spice with therapeutic benefits. In traditional medicine, it is utilized to alleviate symptoms of dizziness, asthma, chronic indigestion, flu, fever, chills, and migraine (Gorgani et al., 2017). Piperine, the primary active compound found in black pepper, has been extensively researched in recent years due to its relatively low side effects and a broad range of physiological activities, including antioxidant, antimicrobial, anti-inflammatory, anticarcinogenic, and cholesterol-lowering effects (Vijayakumar and Nalini, 2006; Shityakov et al., 2019). Other benefits of piperine include promoting blood circulation, stimulating appetite, and producing saliva and digestive secretions in poultry (Ogbuewu et al., 2020). Although the pharmaceutical activities of turmeric and black pepper in humans and various experimental animals have been well-documented, there have been limited studies on the effects of these additives on poultry as natural growth promoters.

Given the pharmacological properties of turmeric and black pepper documented in previous *in vitro* studies, the current research postulated that these phytogenic products would work synergistically to enhance each other’s beneficial effects. Therefore, the present study aimed to investigate the impact of turmeric and black pepper powder, either separately or in combination, on the growth performance, gastrointestinal microbial population, intestinal morphology, blood metabolites, meat quality, and fatty acid profile of Japanese quails. Additionally, the study sought to assess the potential of these additives as alternatives to antibiotics.

## Materials and methods

### Plant material and extraction procedures

Black pepper seeds and turmeric rhizomes were purchased from a local market in Gorgan, Iran. The samples were cleaned, dried, ground to a fine powder, and extracted with 80% ethanol by stirring for 1 h at room temperature. The extraction process was repeated thrice. The sample-to-solvent ratio was 1:10 (w/v). The extracts were filtered using Whatman No. 1 filter paper and concentrated using a vacuum rotary evaporator (Buchi, Switzerland). The final extracts were freeze-dried and stored at −18°C until use.

### Determination of total phenolic contents

The total phenolic content (TPC) of the samples was determined using the Folin–Ciocalteu method, as described by Singleton et al. (1999). Briefly, 50 µL of black pepper and turmeric extract (concentration of 1 mg/mL in methanol) was mixed with 3.95 mL distilled water and 250 µL of Folin–Ciocalteu, respectively. After 4 min, 750 µL of 20% sodium sulfite solution was added and the mixture was allowed to stand for 2 h at room temperature. The absorbance was measured at 760 nm using a UV/Visible Spectrophotometer. Results were expressed as mg Gallic acid equivalent/g of freeze-dried extract (mg GAE/g E).

### Determination of total flavonoid content

Total flavonoid contents (TFC) were determined according to the method described by Cottica. (2015). Briefly, 0.25 mL of 5% aluminum chloride (ethanolic) was mixed with 0.5 mL black pepper and turmeric extract (concentration of 1 mg/mL in methanol). Then, 4.25 mL of methanol was added to the mixture, and absorbance was read at 420 nm after 30 min at room temperature. Total flavonoids were expressed as mg Quercetin equivalent/g of freeze-dried extract (mg QE/g E).

### Antioxidant evaluation (DPPH free radical-scavenging assay)

The antioxidant capacity of the extracts was measured following the method described by Mensor et al. (2001) using the 1,1-diphenyl-2-picrylhydrazyl (DPPH). A volume of 200 µL of extracts (concentration of 1 mg/mL in methanol) was added to 80 µL 0.3 mM methanolic DPPH solution. The mixture was vortexed for 1 min and then allowed to stand in the dark for 30 min at room temperature. The absorbance of the solutions was measured at 517 nm using an ELISA reader (ELx800; BioTek Instruments, Winooski, VT, United States). The antioxidant activity (% inhibition) was calculated according to the following equation:

$$\%\;inhibition=\left(1\;–\;A_{extract}\;/\;A_{control}\right)\times 100$$

where A_control_ represents absorbance of the methanol solution of DPPH without the sample, and A_sample_ is absorbance of the methanol solution of DPPH with extract solution.

### Quails, experimental design, and diets

In a 35-day study, 500 1-day-old Japanese quails were procured from a local commercial hatchery. On arrival, quail chicks were weighed and randomly assigned to one of the five experimental diets with five replicate pens (20 birds per pen) in a completely randomized design. The birds were housed in wire-floored pens (60 cm × 55 cm × 40 cm) in an environmentally controlled room with continuous light and were allowed *ad libitum* access to feed and water throughout the experiment. Upon the arrival of the chicks, the temperature was set at 35°C for the first 3 days and then gradually decreased by 2.5°C per week until it reached 22.5°C. Nutritional requirements of the growing Japanese quails were adopted from NRC (1994) tables, and a basal diet was formulated (Table 1). The quail chicks were fed one of five experimental diets starting from day 1. These included: 1) a corn-soybean basal diet without feed additives (CON), 2) the basal diet with 0.2% antibiotic flavomycin added as an antibiotic growth promoter (AGP), 3) the basal diet with 0.5% turmeric powder added (TUP), 4) the basal diet with 0.5% black pepper powder added (BPP), and 5) the basal diet with a mixture of 0.5% turmeric powder and 0.5% black pepper powder added (MIX).

**TABLE 1:** Ingredients and nutrient contents of the basal diet of Japanese quail.

Items	%
Ingredient	
Maize	55.21
Soybean meal	23.69
Maize gluten meal	10
Fish meal	5.25
Soybean oil	2.84
CaCO3	0.93
Di-calcium phosphate	0.84
Salt	0.23
Vitamin premix^a^	0.25
Mineral premix^b^	0.25
_DL_-methionine	0.03
_L_-lysine	0.36
_L_-threonine	0.13
Calculated composition	
Metabolize energy, kcal/kg	2,900
Crude protein, %	24
^c^SID-Lysine, %	1.27
SID-Methionine, %	0.45
SID-Methionine + cystine, %	0.77
SID-Arginine	1.22
SID-Threonine	0.75
Calcium, %	0.80
Phosphorus (available), %	0.30
Sodium, %	0.15

^a^ Supplied per kg of diet: 1.8 mg all-trans-retinyl acetate; 0.02 mg cholecalciferol; 8.3 mg alphatocopheryl acetate; 2.2 mg menadione; 2 mg pyridoxine HCl; 8 mg cyanocobalamin; 10 mg nicotine amid; 0.3 mg folic acid; 20 mg D-biotin; 160 mg choline chloride.

^b^ Supplied per kg of diet: 32 mg Mn (MnSO_4__H_2_O); 16 mg Fe (FeSO_4__7H_2_O); 24 mg Zn (ZnO); 2 mg Cu (CuSO_4__5H_2_O); 800 µg I (KI); 200 µg Co (CoSO_4_); 60 µg Se (NaSeO_3_).

^c^ SID: standardised ileal digestibility.

### Sampling and measurements

The individual body weight of quails was measured at d 1, 21, and 35, and the mean was calculated for each cage. Feed intake (FI) was recorded on a cage basis. The body weight gain (BWG), FI, and feed conversion ratio (feed: gain, FCR) were calculated for different periods (1–21, 21–35 days), and the total period (1–35 days).

On day 35, 2 birds with body weight close to the average of the cage were selected from each replicate cage. Blood samples were collected from the brachial vein into non-heparinized tubes and centrifuged at 2,500 g for 15 min at 4°C to obtain serum. The serum concentrations of aspartate aminotransferase (AST), alanine aminotransferase (ALT), alkaline phosphatase (ALP), total cholesterol, and triglycerides were determined using an automatic biochemical analyzer (Clima, Ral. Co, Spain), and commercial laboratory kits (Pars Azmoon Kits; Pars Azmoon, Tehran, Iran).

After blood sample collection, the same birds were euthanized by cervical dislocation to evaluate carcass characteristics, meat quality, the crop and ileum’s microbial population, and the small intestine’s morphological characteristics. After removing the feathers, carcass weight, breast, thighs, cloacal bursa, liver, spleen, heart, and abdominal fat were weighed. Then their weights were expressed as a percentage of live body weight.

For histological measurements, a 3 cm segment was first removed from the middle point of the duodenum, jejunum, and ileum; then flushed with distilled water and fixed in 10% neutral buffered formalin for 48 h. The fixed samples were dehydrated, cleared, and embedded in paraffin. Tissue samples (5 μm tickness) were cut by a microtome, stained with haematoxylin-eosin, and scanned using a light microscop. The morphological parameters including villus height (VH: from the top of the villus to the top of the lamina propria), crypt depth (CD: the depth of the invagination between adjacent villi), and villus width (VW: at the middle point of the villus) were measured on each slide. A total of 10 intact villi and crypts were randomly selected in each sample. The ratio of VH to CD was calculated. The ratio of VH to CD was calculated (Jazi et al., 2019). The villus surface area was calculated as 2π × (VW/2) × VH.

To estimate the lactic acid bacteria (LAB) and coliform count in crop and ileum sections, 1 g of crop and ileum contents (separately) was diluted serially in 0.9% sterile saline solution. Afterward, 100 μL of each dilution was plated onto MRS agar (Merck, Germany) and violet red bile agar (Merck, Germany) plates to enumerate the LAB and coliforms, respectively. Plates were then incubated at 37°C for 24–48 h anaerobically (Jazi et al., 2018a). To measure pH, 1 g of each bird’s fresh crop and ileum contents was collected, and the pH was measured using a portable pH meter as per the method by Shabani et al. (2019).

Breast meat samples of euthanized birds were also collected, cut into small pieces, and then stored in the refrigerator at 4°C in the dark for 7 days. The samples’ pH value, malondialdehyde (MDA), meat color, and water-holding capacity (WHC) were analyzed for storage days 1 and 7. The pH value was determined in homogenates prepared by mixing 10 g of sample with 90 mL of distilled water, and a reading was taken with a digital pH meter (NWKbinar pH, K-21, Landsberg, Germany). The meat color of samples was assessed at three different locations across the muscles by using a Laovibond Tintometer Cam-System 500 (Amesbury, United Kingdom) and expressed as lightness (L*), redness (a*), and yellowness (b*). The water holding capacity was measured by centrifuging 1 g of the muscles placed on a round plastic plate in a tube for 4 min at 1,500 g and drying at 70°C (Castellini et al., 2002). The MDA level was measured by 2-TBA, the absorbance change at 532 nm was monitored by a spectrophotometer, and data were expressed as nanomole per mg of protein for muscle samples.

The breast muscle specimens were also evaluated for fatty acid analysis. First, the total lipids from thigh tissue were extracted using a chloroform and methanol solution to prepare fatty acid methyl esters. Then, fatty acid methyl esters were analyzed with a Hewlett-Packard 5,890 gas chromatograph equipped with an autosampler, a flame ionization detector, and a fused silica capillary column (30 m × 0.25 mm i.d; Chrompack, Middelburg, Netherlands). The initial oven temperature was 110°C, held for 1 min, then increased to 190°C at 150°C/min and held for 55 min, then increased to 230°C at 5°C/min and held for 5 min. Helium at 30 psi was used as the carrier gas at the flow rate of 0.5 mL/min. Identification of fatty acid methyl esters was made by comparing their retention times with the authentic external standards and obtained values were expressed as a percentage of fatty acid methyl esters or as g/100 g of sample (Apperson and Cherian, 2017).

### Statistical analysis

The Student’s t-test was used to assess the TPC, TFC, and antioxidant capacity data, and the variability was expressed as standard error. Data obtained during the feeding period of quail chicks were checked for normality and then analyzed using the GLM procedures of SAS statistical software (SAS, 2010) version 9.4, as a completely randomized design. Tukey’s test was used to compare significant differences among the means, and the level of probability less than 0.05 were considered statistically different. Each pen was the experimental unit for all variables studied.

## Results

### Estimation of bioactive compounds

Table 2 presents the mean TPC, TFC, and DPPH values in the black pepper and turmeric extracts. The methanol extract of turmeric was found to have a higher (*p* < 0.001) TPC (about 83.24%) and TFC (about 93.73%) than the black pepper extract. In addition, the turmeric extract showed higher (*p* < 0.001) DPPH radical scavenging activity (about 90%).

**TABLE 2:** Total phenolic, flavonoid content, and antioxidant activity for black pepper and turmeric extracts.^a^

	TPC^b^	TFC^c^	DPPH^d^
Black pepper	32.20 ± 2.03^b^	19.79 ± 0.48^b^	63.19 ± 0.90^b^
Turmeric	192.18 ± 2.49^a^	315.73 ± 2.45^a^	76.83 ± 0.71^a^
*p*-value	<0.0001	<0.0001	<0.0001

^a^ Results are reported as means of 6 replicates ± standard error on a dry weight basis.

^b^ TPP, Total phenolic content (as mg gallic acid equivalent/g of freeze-dried extract).

^c^ TFC, Total flavonoid content (as mg quercetin equivalent/g of freeze-dried extract).

^d^ DPPH, Antioxidant activity evaluated by free radical scavenging activity (as % inhibition). DPPH, scavenging capability of vitamin C (as control) was 95.36%.

### Growth performance

The effects of dietary treatments on the growth performance of growing Japanese quails are presented in Table 3. The tested feed additives did not affect the FI during the starter period. However, the FI during the grower and entire experimental periods in birds fed the CON and BPP diets was lower (*p* < 0.007) than in the other treatments. Quail chicks fed diets supplemented with AGP and TUP had a higher (*p* = 0.01) BWG than those in the CON, BPP, and MIX groups during the starter period. In addition, BWG in the AGP and TUP groups was significantly greater than in the other treatments during the grower (*p* = 0.003) and entire experimental periods (*p* = 0.007). The FCR during the starter and grower periods was significantly better (*p* = 0.02) in birds fed the experimental diets than in the CON diet. During the entire rearing period, quail chicks fed AGP and TUP diets had better (*p* = 0.02) FCR than the other treatments. At all periods, birds fed AGP exhibited a lower FCR than those in the CON group. The results also showed that birds in the TUP group had a numerically lower FCR than those in the CON, BPP, and MIX groups.

**TABLE 3:** Effects of TUP, BPP, and MIX on growth performance of Japanese quails.

	Treatment^a^						
Item^b^	CON	AGP	TUP	BPP	MIX	SEM	*p*-value
1–21 days							
BWG, g	90.10^b^	102.55^a^	100.36^ab^	91.24^b^	93.78^ab^	2.56	0.013
FI, g	224.33	239.98	240.78	225.08	232.80	7.28	0.361
FCR, g/g	2.49^a^	2.34^b^	2.40^ab^	2.46^ab^	2.48^ab^	0.03	0.020
21–35 days							
BWG, g	70.27^b^	83.37^a^	79.27^ab^	71.46^b^	72.93^b^	2.21	0.003
FI, g	318.23^b^	360.48^a^	352.82^ab^	319.60^b^	327.77^ab^	8.52	0.007
FCR, g/g	4.53^a^	4.32^b^	4.45^ab^	4.47^ab^	4.49^ab^	0.04	0.061
1–35 days							
BWG, g	160.38^c^	185.92^a^	179.63^ab^	162.71^c^	166.72^bc^	3.72	0.007
FI, g	542.56^b^	600.48^a^	593.60^ab^	544.68^b^	560.57^ab^	12.17	0.009
FCR, g/g	3.38^a^	3.22^b^	3.30^ab^	3.35^a^	3.36^a^	0.02	0.015

^a–c^Means with different superscripts in each row are statistically different (*p* < 0.05).

Data represent means of 5 replicates of 20 quails per treatment.

^a^ CON, control; AGP, antibiotic growth promoter; TP, turmeric powder; BPP, black pepper powder; MIX, TP plus BPP.

^b^ BWG, body weight gain; FI, feed intake; FCR, feed conversion ratio.

### Carcass characteristics and meat quality

The effects of experimental diets on carcass characteristics and meat quality of Japanese quail are summarized in Table 4. The breast yield in birds fed with the CON diet was less (*p* = 0.03) than in the other groups. All the tested feed additives except AGP reduced (*p* = 0.005) abdominal fat percentage. However, the treatments did not affect the relative weights of the carcass, thigh, liver, spleen, cloacal bursa, and heart.

**TABLE 4:** Effects of TUP, BPP, and MIX on carcass characteristics and meat quality of breast muscle in Japanese quails at 35 days of age.

	Treatment^a^						
Item^b^	CON	AGP	TUP	BPP	MIX	SEM	*p*-value
Carcass, % LBW	69.41	70.66	69.54	70.26	69.88	0.72	0.730
Breast, % LBW	24.05^b^	26.37^a^	25.40^ab^	25.12^ab^	25.31^ab^	0.41	0.025
Thigh, % LBW	17.74	18.17	18.07	17.64	17.82	0.83	0.981
Abdominal fat, % LBW	1.17^ab^	1.64^a^	0.63^b^	0.74^b^	0.59^b^	0.14	0.005
Liver, % LBW	3.03	2.96	2.84	3.00	3.20	0.21	0.830
Spleen, % LBW	0.05	0.05	0.04	0.05	0.05	0.01	0.675
Cloacal bursa, % LBW	0.15	0.16	0.15	0.17	0.18	0.01	0.321
Heart, % LBW	0.81	0.72	0.63	0.88	0.94	0.86	0.698
pH_1 d_	6.09	6.11	6.16	6.18	6.15	0.01	0.123
pH_7 d_	5.97^c^	5.95^c^	6.13^ab^	6.10^b^	6.20^a^	0.01	0.012
WHC_1 d,_%	66.81	66.54	66.94	67.26	67.39	0.02	0.104
WHC_7 d_,%	61.94^b^	61.63^b^	62.42^ab^	62.50^ab^	63.25^a^	0.01	0.033
L*_1 d_	45.47	45.59	44.34	45.38	44.04	0.28	0.365
L*_7 d_	48.04^a^	48.12^a^	46.53^b^	47.20^ab^	46.12^b^	0.33	0.015
a*_1 d_	6.21	6.18	6.30	6.59	6.61	0.19	0.401
a*_7 d_	7.05	7.22	7.87	7.70	7.98	0.22	0.687
b*_1 d_	25.76	25.89	25.63	25.14	25.80	0.45	0.614
b*_7 d_	27.05	27.13	26.33	26.90	25.92	0.50	0.321

^a-c^Means with different superscripts in each row are statistically different (*p* < 0.05).

Data are means of 5 replicate pens, 2 birds per pen.

^a^ CON, control; AGP, antibiotic growth promoter; TUP, turmeric powder; BPP, black pepper powder; MIX, TUP plus BPP.

^b^ LBW, live body weight; WHC, water holding capacity; L*, lightness; a*, redness; b*, yellowness.

**FIGURE 1:**
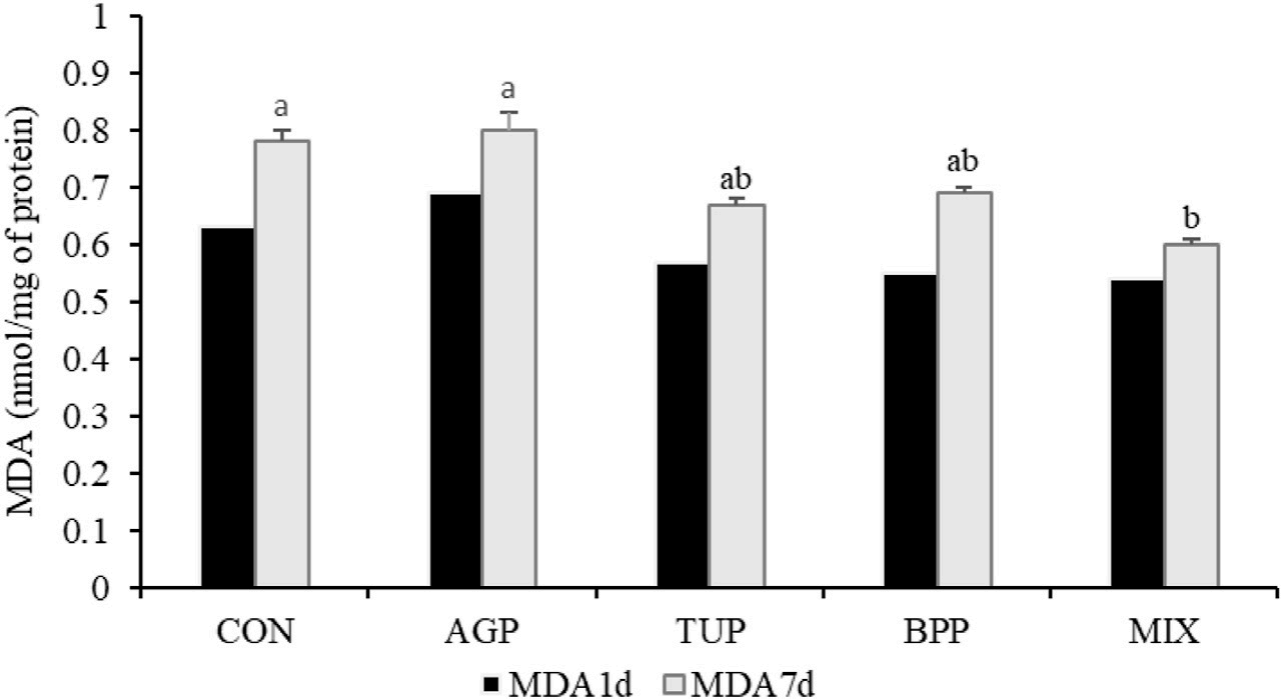
Effects of TUP, BPP, and MIX on malondialdehyde (MDA) concentration of breast muscle in Japanese quails at 35 days of age. Data are means of 5 replicate pens, 2 birds per pen. CON, control; AGP, antibiotic growth promoter; TUP, turmeric powder; BPP, black pepper powder; MIX, TUP plus BPP. MDA1d and MDA7d, the content of malondialdehyde 1 and 7 days after storage. Bars with different letters (a, b) statistically differ (*p* < 0.05).

The breast muscle pH and WHC in the birds fed phytogenic additives, particularly in the MIX group, increased (*p* = 0.04; Table 4) after 7 days of refrigerated storage compared to the other groups. Diets supplemented with TUP and MIX decreased (*p* = 0.02) the lightness index in meat samples after 7 days of refrigerated storage compared to the other diets. In addition, after 7 days storage period, the MDA concentration in the MIX group was lower (Figure 1; *p* = 0.02) than in the CON and AGP groups. However, the experimental diets did not affect pH (1 day after storage), WHC (1 day after storage), lightness (1 day after storage), redness (one and 7 days after storage), yellowness (one and 7 days after storage), and the MDA concentration (1 day after storage) of the breast meat.

### Gut microbiota and pH

Table 5 depicts the effects of experimental diets on pH and microbial population in crop and ileum of Japanese quails. The coliform population was higher (*p* < 0.001) in the crop and ileum, while the LAB population was lower (*p* = 0.008) in the ileum section of birds fed the CON diet compared to the other groups. In addition, all the tested feed additives significantly reduced (*p* < 0.001) the pH of the ileum. Diets containing TUP and MIX showed the best performance in improving the microbiota balance. However, the treatment groups did not affect the pH and LAB population in the crop.

**TABLE 5:** Effects of TUP, BPP, and MIX on gastrointestinal microbiota composition and pH value in Japanese quails at 35 days of age.

	Treatment^a^						
Item	CON	AGP	TUP	BPP	MIX	SEM	*p*-value
Crop							
pH	5.05	5.06	4.97	4.91	5.05	0.06	0.484
LAB^b^	7.72	7.69	8.06	7.99	7.92	0.09	0.071
Coliforms	6.36^a^	5.01^b^	5.74^ab^	5.63^ab^	5.52^b^	0.19	<.001
Ileum							
pH	6.67^a^	6.30^days^	6.43^bc^	6.50^b^	6.37^cd^	0.03	<.001
LAB	7.08^b^	7.55^a^	7.56^a^	7.27^ab^	7.50^a^	0.08	0.008
Coliforms	7.11^a^	5.98^b^	6.12^b^	6.60^ab^	6.14^b^	0.19	<.001

^a-d^Means with different superscripts in each row are statistically different (*p* < 0.05).

Data are means of 5 replicate pens, 2 birds per pen.

^a^ CON, control; AGP, antibiotic growth promoter; TUP, turmeric powder; BPP, black pepper powder; MIX, TUP plus BPP.

^b^ LAB, lactic acid bacteria.

### Intestinal morphology

The morphological characteristics of the small intestine in growing Japanese quails are presented in Table 6. The VH to CD ratio in the duodenum was greater (*p* = 0.02) in the birds fed the tested additives, particularly the MIX diet, than in the CON diet. In the jejunum and ileum, the VH and VH to CD ratios were increased (*p* < 0.05) by supplementation with AGP, TUP, BPP, and MIX compared to the CON diet. In addition, the tested feed additives, particularly MIX and AGP, reduced (*p* = 0.02) CD in the jejunum. However, gut morphology assessments did not reveal any other differences in the morphometric parameters in the birds fed dietary supplements’ duodenum, jejunum, and ileum.

**TABLE 6:** Effects of TUP, BPP, and MIX on intestinal morphometric analysis in Japanese quails at 35 days of age.

	Treatment^a^						
Item	CON	AGP	TUP	BPP	MIX	SEM	*p*-value
Duodenum							
Villus height (VH), µm	776.89	806.26	769.97	784.85	825.67	24.10	0.242
Crypt depth (CD), µm	117.15	104.01	113.50	112.02	104.38	5.65	0.191
VH:CD	5.88^b^	7.03^ab^	6.79^ab^	6.68^ab^	7.83^a^	0.36	0.022
Villus width, µm	141.20	153.73	145.10	152.17	153.12	11.08	0.793
Villus surface area, mm^2^	0.35	0.37	0.35	0.34	0.39	0.03	0.540
Jejunum							
Villus height (VH), µm	610.02^c^	655.48^a^	629.82^b^	624.12^bc^	628.08^b^	3.81	<.001
Crypt depth (CD), µm	120.84^a^	111.30^b^	113.44^ab^	119.67^ab^	111.06^b^	2.12	0.015
VH:CD	5.05^c^	5.89^a^	5.56^ab^	5.22^bc^	5.65^ab^	0.10	0.001
Villus width, µm	159.88	159.78	161.07	160.33	160.27	4.20	0.990
Villus surface area, mm^2^	0.30	0.33	0.32	0.31	0.31	0.09	0.474
Ileum							
Villus height (VH), µm	429.16^c^	495.66^a^	494.04^a^	436.80^b^	488.18^a^	5.38	<.001
Crypt depth (CD), µm	105.58	112.62	114.76	110.69	113.72	2.61	0.162
VH:CD	4.07	4.40	4.31	4.20	4.29	0.07	0.063
Villus width, µm	91.34	88.13	89.96	80.80	88.65	5.90	0.746
Villus surface area, mm^2^	0.12	0.13	0.14	0.11	0.13	0.01	0.425

^a-d^Means with different superscripts in each row are statistically different (*p* < 0.05).

Data are means of 5 replicate pens, 2 birds per pen.

^a^ CON, control; AGP, antibiotic growth promoter; TUP, turmeric powder; BPP, black pepper powder; MIX, TUP plus BPP.

### Serum biochemical parameters

Data on blood serum biochemistry are shown in Table 7. Feeding quails with experimental diets including TUP, BPP, and MIX decreased (*p* = 0.001) serum concentration of cholesterol compared to the CON diet. Dietary treatments did not affect triglycerides, ALP, ALT, and AST serum concentrations.

**TABLE 7:** Effects of TUP or/and BPP on serum biochemical parameters in Japanese quails at 35 days of age.

	Treatment^a^						
Item^b^	CON	AGP	TUP	BPP	MIX	SEM	*p*-value
Cholesterol, mg/dL	131.25^a^	128.25^ab^	109.50^c^	115.75^c^	117.75^bc^	2.54	0.001
Triglycerides, mg/dL	67.60	79.80	63.40	63.00	63.80	7.15	0.495
ALP, U/L	366.10	390.63	375.10	379.02	334.41	24.31	0.563
ALT, U/L	6.06	6.39	6.21	6.16	6.03	0.39	0.972
AST, U/L	136.81	143.06	136.40	138.14	136.53	3.42	0.621

^a-c^Means with different superscripts in each row are significantly different (*p* < 0.05).

Data are means of 5 replicate pens, 2 birds per pen.

^a^ CON, control; AGP, antibiotic growth promoter; TUP, turmeric powder; BPP, black pepper powder; MIX, TUP, plus BPP.

^b^ ALP, alkaline phosphatase; ALT, alanine aminotransferase; AST, aspartate aminotransferase.

### Fatty acid profile

The effect of the experimental diets on fatty acid content in the breast muscle of Japanese quails is shown in Table 8. Birds fed diets containing phytogenic products (TUP, BPP, and in particular MIX) had higher (*p* ˂ 0.05) contents of C16:1, C18:1, C18:2, C20:5, MUFA, PUFA, and PUFA/SFA ratio and lower (*p* ˂ 0.001) concentrations of C16:0 and SFA compared to the CON diet. The ratio of hypocholesterolaemic to hypercholesterolaemic (H/H) fatty acid increased (*p* = 0.001) in response to dietary TUP and MIX. However, no significant differences were found between the treatment groups in the contents of C14:0, C18:0, C20:0, C18:3 n-3, C18:3 n-6, C20:1, C20:2, C20:2 n-3, C20:2 n-6, C20:4, C20:5, C20:6, and n-3: n-6 ratio in the breast muscle.

**TABLE 8:** Effects of TUP or/and BPP on the fatty acid profile of breast muscle in Japanese quails at 35 days of age.

	Treatment^a^						
Fatty acid (%)	CON	AGP	TUP	BPP	MIX	SEM	*p*-value
C14:0	0.60	0.62	0.64	0.66	0.61	0.01	0.376
C16:0	26.33^a^	24.34^ab^	23.43^b^	23.73^b^	22.40^b^	0.37	<.001
C18:0	9.25	9.16	8.64	8.99	8.64	0.10	0.178
C20:0	0.67	0.65	0.68	0.74	0.72	0.01	0.438
C16:1 n-7	3.47^b^	4.40^ab^	4.61^a^	4.57^a^	4.63^a^	0.14	0.021
C18:1 n-9	31.39^ab^	31.17^b^	32.27^ab^	32.02^ab^	32.52^a^	0.15	0.031
C18:2 n-6	16.84^b^	17.86^ab^	17.91^ab^	17.71^ab^	18.33^a^	0.14	0.010
C18:3 n-3	0.79	0.81	0.83	0.86	0.84	0.01	0.139
C18:3 n-6	0.35	0.34	0.35	0.36	0.37	0.01	0.810
C20:1 n-9	0.65	0.66	0.71	0.65	0.70	0.01	0.734
C20:2 n-6	0.42	0.44	0.45	0.42	0.48	001	0.231
C20:3 n-3	0.10	0.28	0.24	0.09	0.27	0.05	0.716
C20:3 n-6	2.23	2.25	2.20	2.61	2.17	0.05	0.080
C20:4 n-6	2.59	2.80	2.76	2.41	2.77	0.04	0.092
C20:5 n-3	0.72^a^	0.69^ab^	0.68^ab^	0.61^b^	0.72^a^	0.01	0.021
C22:5 n-3	0.64	0.64	0.69	0.71	0.72	0.01	0.102
C22:6 n-3	2.65	2.84	2.79	2.80	3.03	0.04	0.159
Saturated fatty acid (SFA)	36.86^a^	34.78^ab^	33.41^bc^	34.13^bc^	32.38^c^	0.39	<.001
Monounsaturated fatty acid (MUFA)	35.11^b^	35.57^ab^	36.88^a^	36.60^ab^	37.16^a^	0.23	0.006
Polyunsaturated fatty acid (PUFA)	28.02^b^	29.64^a^	29.70^a^	29.26^ab^	30.43^a^	0.23	0.004
PUFA/SFA	0.76^b^	0.85^ab^	0.89^a^	0.86^a^	0.94^a^	0.01	<.001
n-6/n-3^b^	4.59	4.49	4.48	4.64	4.32	0.06	0.611
H/H^c^	2.08^c^	2.27^bc^	2.41^ab^	2.34^abc^	2.56^a^	0.04	0.001

^a-c^Means with different superscripts in each row are significantly different (*p* < 0.05).

Data are means of 5 replicate pens, 2 birds per pen.

^a^ CON, control; AGP, antibiotic growth promoter; TUP, turmeric powder; BPP, black pepper powder; MIX, TUP, plus BPP.

^b^ n-6/n-3, total omega 6 to total omega 3 fatty acid ratio.

^c^ H/H, hypocholesterolaemic to hypercholesterolaemic fatty acid ratio.

## Discussion

The use of PFAs has become increasingly popular in the post-antibiotic era due to their various biological properties, including antibacterial, antioxidant, and anti-inflammatory effects. Phenolic and flavonoid compounds, which are secondary metabolites of plants, are primarily responsible for their antioxidant and antimicrobial activities. In the current study, turmeric extract’s phenolic and flavonoid contents were higher than black pepper extract’s, resulting in higher DPPH scavenging activity for turmeric. The feeding trial results indicated that quail chicks fed diets supplemented with AGP and TUP had higher body weight gain and lower FCR than the other groups. However, adding BPP to the experimental diets did not enhance growth performance at any trial stage. Likewise, Khan and Ahmad (2022) reported that TUP at a 0.1% inclusion rate increased BWG and reduced FCR in broiler chickens. Yarru et al. (2009) also suggested adding TUP at a 0.5% inclusion rate to diets to improve BWG in aflatoxin-exposed broiler chickens. Furthermore, prior research has demonstrated that adding different PFAs to broiler diets, such as *Pulicaria gnaphalodes* powder and *Achyrabthes japonica* extract, can improve BWG, FCR, nutrient digestibility, gut microbiota, and intestinal morphology (Shirani et al., 2019; Park and Kim, 2020). Plant extracts, spices, and herbs can improve nutrient digestion in poultry by stimulating various digestive functions, such as appetite, saliva secretion, bile acid secretion, and digestive enzyme activity, including lipase, amylase, and protease (Jang et al., 2007; Oso et al., 2019). These outcomes can ultimately contribute to improved growth performance. The improved growth performance in birds fed the TUP diet in the present study was associated with the higher concentrations of phenolic and flavonoid compounds in turmeric extract. Nevertheless, some studies on PFAs in poultry diets have yielded inconsistent results regarding growth performance, such as the report by Jang et al. (2007). The lack of impact of diets containing BPP on growth performance in the current study is likely due to factors like inclusion dosage, BPP composition, active ingredient concentrations, and their biological activity.

In terms of carcass characteristics, breast yield was found to be higher in birds fed AGP, TUP, and MIX than in the other groups, indicating the growth-promoting effect of these feed additives. Furthermore, the present study’s findings demonstrate that using herbal additives (TUP, BPP, and MIX) can reduce abdominal fat in Japanese quails. Similarly, previous studies have demonstrated that dietary supplementation with 100–300 mg/kg turmeric rhizome extract reduces abdominal fat in broiler chickens (Wang et al., 2015). Xie et al. (2019) investigated the effect of curcumin (the primary constituent of turmeric) on the lipid metabolism of broiler chickens and found that curcumin could reduce abdominal fat by decreasing hepatic and plasma lipid concentration and regulating the expression of genes associated with lipogenesis and lipolysis, such as acetyl-CoA carboxylase. According to other studies, active substances present in medicinal plants can also reduce abdominal fat by inhibiting the synthesis of adipose tissue through the modulation of fatty acid transportation (Shirani et al., 2019).

Meat pH is a significant factor that affects meat quality attributes such as color and WHC. A decrease in postmortem pH can result in protein denaturation, leading to paleness and decreased WHC of the meat. The data from this study indicated that the MIX treatment increased the meat sample pH value after 7 days of refrigerated storage compared to the CON and AGP treatments. The WHC of meat is another critical parameter that indicates the muscle tissue’s ability to retain moisture and directly affects meat taste and tenderness. A lower WHC reflects losses in the meat’s nutritional value through released exudates, resulting in tougher and less flavorful meat (Cao et al., 2012). The simultaneous inclusion of TUP and BPP in the diet and their synergistic effect can control oxidation reactions in meat to maintain water storage space between myofibrils and increase WHC in meat (Huff-Lonergan and Lonergan, 2005). Rajput et al. (2014) also reported that natural antioxidants, such as carotenoids in curcumin, can improve meat’s WHC by modulating the redox state and enhancing the antioxidant capacity in muscle. Meat color is a crucial criterion related to meat freshness and quality, affecting consumer acceptance of meat. It is evaluated by determining the lightness, redness, and yellowness (Niu et al., 2017). According to Jiang et al. (2014), consumers prefer redness; a lower yellowness index indicates less pale meat. In the present study, diets supplemented with TUP and MIX decreased the lightness index in meat samples after 7 days of refrigerated storage compared to the other diets. Niu et al. (2017) suggested that the decrease in the lightness index might be related to the increased antioxidant activity of PFAs, which protects cells from damage and prevents cell juice extravasation, ultimately decreasing light reflection.

The MDA concentration in tissue indirectly mirrors lipid peroxidation, which is the outcome of attenuated antioxidant protection (Jazi et al., 2020). Long-term storage is one of the reasons for the increased lipid peroxidation in meat after slaughter (Aziza et al., 2010). In the current study, lower MDA content in breast muscle during 7 days of storage at 4°C was observed when quails were fed the MIX diet. Thus, the simultaneous inclusion of TUP and BPP may provide a more efficient free radical scavenging activity in birds. Similarly, improvements in the lipid peroxidation of meat have been reported in several studies when herbal additives rich in phenolic compounds or flavonoids were added to the diet (Aziza et al., 2010; Cao et al., 2012).

The current study found that the tested feed additives reduced pH and coliforms in the crop and ileum while increasing the LAB population in the ileum of birds. The growth inhibition of coliforms by TUP and BPP was similar to that of AGP. Curcumin possesses high antibacterial activity against *Clostridium difficile*, as shown by an *in vitro* study by Mody et al. (2020). Additionally, the administration of curcumin in humans has been reported to markedly shift the ratio of beneficial and pathogenic bacteria by enhancing the population of *lactobacilli*, *bifidobacteria*, and butyrate-producing bacteria and decreasing the number of *enterococci* and *enterobacteria*, as reported by Zam (2018). Moreover, several *in vivo* studies have demonstrated the potent antibacterial activity of black pepper essential oil against pathogenic *Escherichia coli* and *Staphylococcus*. (Zhang et al., 2017; Abdallah and Abdolla, 2018). Some studies with Japanese quails (Mehri et al., 2015) and broiler chickens (Chowdhury et al., 2018) indicate *in vivo* antibacterial activity of different PFAs (peppermint and cinnamon) against intestinal pathogenic microbes such as coliform and *E. coli*. One of the critical features of PFAs is their hydrophobicity, allowing them to easily enter the bacterial cell membrane, leading to cell membrane disintegration, leakage of intracellular material, reduction of proton force, and, eventually, death of the bacterial cell (Zeng et al., 2015; Barbarestani et al., 2020). Therefore, reducing the population of pathogenic bacteria, such as coliforms within the gastrointestinal tract, may increase the number of beneficial bacteria, such as LAB. Another possible explanation for the positive effect of PFAs on the growth of beneficial bacteria is that LAB can metabolize phenolic compounds as nutritional substrates, suggesting the prebiotic-like impact of phenolic compounds (Iqbal et al., 2020).

Intestinal epithelial integrity and structure play an important role in nutrient digestion, absorption, and overall gut health (Soumeh et al., 2019). Intestinal villi are the leading site for absorption of nutrients; therefore, longer villi may indicate a greater surface area, which in turn can enhance nutrient absorption capacity, while deeper crypt may suggest fast cellular turnover and regeneration processes of tissue due to damage that is induced by pathogens (Jazi et al., 2018b). Therefore, VH: CD can reflect digestive and absorptive capacities. The present study showed that the MIX treatment group had a higher VH: CD ratio in the duodenum, while the AGP, TUP, and MIX groups had higher VH and VH: CD ratio in the jejunum. The tested feed additives increased the ileum’s VH compared to the control group. Improving the intestinal mucosal structure in response to dietary PFAs may be linked to an increased population of beneficial bacteria, which can produce antibacterial compounds and compete with harmful pathogens like coliforms. This can prevent their colonization and minimize their negative impact on the intestinal structure (Jazi et al., 2017). Feeding broiler chickens with PFAs may also stimulate mucus secretion into the intestine, which can act as a dynamic protective surface and prevent the adherence of pathogens to intestinal epithelial cells (Cornick et al., 2015; Zeng et al., 2015). Furthermore, the antioxidant activity of PFAs may protect villi from oxidative stress caused by digestive processes (Zeng et al., 2015). The present study found that combining TUP and BPP in the diet improved the population of gastrointestinal microbiota and intestinal morphology more effectively, indicating synergistic effects between TUP and BPP.

The serum concentrations of cholesterol and triglycerides can reflect lipid metabolism (Sharifi et al., 2023). The current study revealed that the tested feed additives reduced the concentration of cholesterol. Kim and Kim (2010) reported a significant reduction in serum cholesterol levels in rats fed an excessive fat diet supplemented with curcumin, possibly due to increased expression of cholesterol 7α-hydroxylase, a rate-limiting enzyme in bile acid formation from cholesterol. Xie et al. (2019) suggested that curcumin reduces cholesterol by inhibiting ATP-citrate lyase, a key enzyme in fatty acid synthesis that converts citrate to acetyl CoA. Piperine, the active compound in black pepper, is known to reduce plasma and liver cholesterol by changing cholesterol transporter proteins and reducing lipogenic gene expression (Tunsophon and Chootip, 2016). Shirani et al. (2019) suggested that the hypocholesterolemic effect of PFAs may result from the active ingredient’s inhibitory effects on the 3-hydroxy-3-methyl glutaryl-CoA enzyme, a key enzyme for cholesterol biosynthesis. Serum levels of ALT, ALP, and AST serve as indicators of liver health and functionality, as their concentrations increase when liver cells are destroyed (Mohebodini et al., 2019). However, in the present study, the experimental treatments did not affect the concentrations of these enzymes and therefore had no negative impact on the liver.

Researchers have recently focused on modifying fatty acid compounds in poultry meat, as the level of SFA are correlated to hypercholesterolemia and coronary heart disease (Ahmed et al., 2015; Shirani et al., 2019). Our results demonstrated that dietary supplementation with TUP, BPP, and MIX increased the concentrations of total MUFA, PUFA, and PUFA/SFA ratio while decreasing total SFA content. However, the MIX diet had a more pronounced effect on the meat fatty acid profile, possibly due to the synergistic effects. Similar observations were made in broiler chickens fed phytogenic products containing TUP by Daneshyar et al. (2011). It has been suggested that palmitic acid (C16:0) often raises blood cholesterol levels, while oleic acid (C18:1) has the opposite effect on blood cholesterol. Additionally, PUFA may decrease abdominal fat deposition by enhancing the expression of acyl-CoA oxidase (the main enzyme of the beta-oxidation process) (Crespo and Esteve-Garcia, 2001; 2002). The decrease in SFA in the meat of birds may be related to an increase in the amount of oleic acid (C18:1) and MUFA, given that stearic acid (C18:0) is converted to oleic acid (C18:1) more rapidly (Ahmed et al., 2015). In addition, the enhanced PUFA concentration may be due to the improvement of the antioxidant defense system, which could protect PUFA oxidation and cause the production of stable products (Mohebodini et al., 2021). The PUFA: SFA and n-6: n-3 ratios are typically used to assess the nutritional value of meat. Ahmed et al. (2015) suggested that meat with a PUFA: SFA ratio less than 0.4 and n-6: n-3 ratio greater than 5 is considered unfavorable because they may increase cholesterolemia. In the current study, although the n-6: n-3 ratio was not affected by the experimental treatments, the PUFA: SFA ratio in birds fed the additives was higher than the minimum recommended values, indicating a positive effect of the tested PFAs on meat quality. In addition to the above indices, a more comprehensive approach to the nutritional evaluation of meat could involve using indices based on the functional effects of fatty acids, such as the HH ratio (Santos-Silva et al., 2002). The highest HH index values were recorded in birds fed the MIX diet, indicating a better nutritional value of meat in this group. Therefore, these findings suggest that consuming these meats may lower humans’ risk of coronary disease.

The present study indicates that the simultaneous application of TUP and BPP to improve growth performance is ineffective. However, TUP alone can result in improvements in BWG and FCR. Additionally, because the simultaneous inclusion of TUP and BPP can positively affect the population of gastrointestinal microbiota and intestinal morphology, their combination may be more effective under stressful conditions. Combining TUP and BPP in the diet also improves breast meat quality and shelf life, indicating the synergistic effects between TUP and BPP.

## Data Availability

The raw data supporting the conclusion of this article will be made available by the authors, without undue reservation.
